# Improvement of foaming properties of ovalbumin: Insights into the synergistic effect of preheating and high-intensity ultrasound on physicochemical properties and structure analysis

**DOI:** 10.1016/j.ultsonch.2023.106672

**Published:** 2023-10-31

**Authors:** Zhihui Yu, Li Ma, Binbin Liu, Wenqing Wang, Ziqi Shang, Huichao Dang, Chunyou Liu

**Affiliations:** a College of Food Science and Engineering, Shanxi Agricultural University, Taigu 030801, Shanxi, China; b Department of Food Science and Technology, School of Biological and Chemical Engineering, Guangxi University of Science and Technology, Liuzhou, China

**Keywords:** Ovalbumin, Preheating, High-intensity ultrasound, Foaming properties, Structure

## Abstract

Ovalbumin (OVA), characterized by its high concentration in eggs, possesses remarkable foaming properties. Nevertheless, OVA is highly sensitive to thermal changes and acid-base conditions, substantially hampering its application potential for foaming purposes within the food industry. This experiment aimed to examine the effects of preheating and high-intensity ultrasound (HIU) treatment at different powers on OVA foaming properties and explore the underlying mechanisms. The results revealed that OVA exhibited the highest foaming capacity (31.5 %) and foaming stability (96.7 %) under the treatment condition of 200w + 60°C. Additionally, significant improvements were observed in the content of free sulfhydryl groups (37.27 μmg/g), solution viscosity (142.33 mPa·s), and surface hydrophobicity (37.27 μg BPB) under this condition. The absolute value of the zeta potential (-10.28 mV) was significantly increased in the 200w + 60°C treatment group. Moreover, the polymer dispersity index of OVA (0.6045) was significantly reduced, resulting in improved dispersion than the control group. The structural analysis revealed significant changes in the α-helix and β-sheet content of OVA after treatment at 200w + 60 °C. The X-ray diffraction pattern exhibited sharper peaks, indicating a crystal structure, and the fluorescence peak displayed a slight blue shift along with increased hydrophobicity. Moreover, the preheating and HIU treatment induced a continuous uneven and irregular pore structure in OVA, which ultimately enhanced its foaming properties. In conclusion, the preheating and HIU treatment offers a novel approach to enhance the foaming properties of OVA.

## Highlights

• OVA exhibited the highest foaming capacity and stability at 200w + 60°C.
• Significant improvements in sulfhydryl groups, viscosity, and hydrophobicity at 200w + 60°C.
• Zeta potential and particle size affected by 200w + 60℃ treatment.
• significant changes in the α-helix and β-sheet content of OVA after HIU treatment.
• The preheating and HIU treatment induced a continuous uneven and irregular pore structure in OVA.

## Graphical abstract

**Figure:**
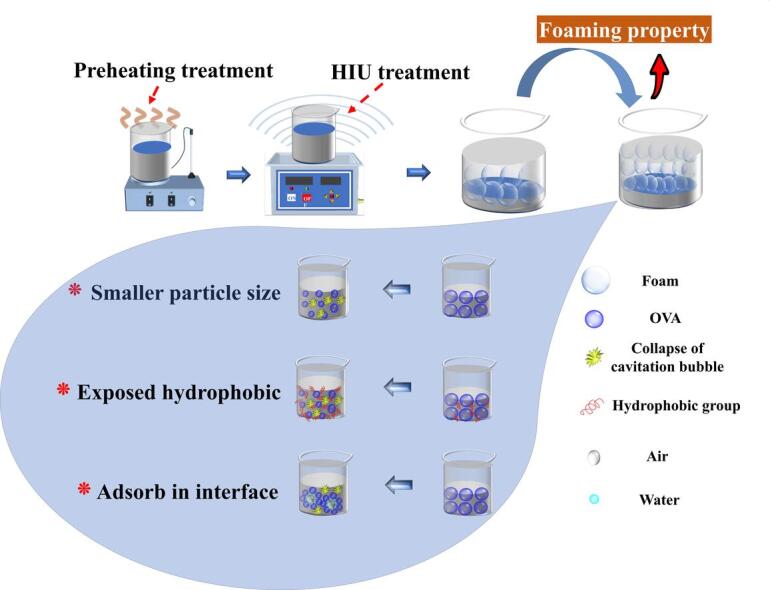

## 1 Introduction

Ovalbumin (OVA), one of the main proteins in egg white, accounts for approximately 54 % to 63 % of the total egg white protein content [1]. OVA possesses an amphiphilic molecular structure and demonstrates interfacial activity in the water/oil phase. When subjected to rapid stirring, it quickly adsorbs to the water–air interface, leading to partial unfolding of its molecules and exposing hydrophobic groups. This process leads to a decrease in interfacial tension and the incorporation of air, leading to the formation of a thin and viscoelastic film and foam at the air–water interface [2]. Hence, the foaming properties of OVA are regarded as one of its most significant characteristics, which encompasses foaming capacity (FC) and foaming stability (FS). This ability depends on the diffusion of protein molecules at the air–water interface and the unfolding of their molecular structure. On the other hand, the stability of the foam relies on the formation of a thick and viscous layer during the stirring process. The formation and stabilization of foam require the presence of protein adsorption layers, and the FC of proteins is directly associated with their capability to diffuse and adsorb at the interface [3]. Several external factors, including physical and chemical factors, can influence the FC and FS of OVA at the air–water interface. Besides, OVA is more sensitive to heat and less stable, making it vulnerable to high temperatures, acids, and bases during food processing [4], [5]. Therefore, modifications are necessary to enhance its foaming properties and increase its value in various applications.

Currently, the main methods of modification of protein include chemical modification, enzymatic modification, and physical modification [6]. However, chemical modification and enzymatic modification may leave chemical reagent residues and relatively poor stability, which limits their application in protein modification [7]. In comparison, physical modification offers the advantage of minimal loss of nutritional value in proteins and has the benefits of low processing cost, simplicity, and high safety [8]. Physical treatment methods currently used include ultrasound, high-pressure pulse, and ultra-high pressure. Among them, high-intensity ultrasound (HIU) treatment has attracted wide attention for its environmental friendliness and energy efficiency [9]. It utilizes high-frequency sound waves to modify proteins, reducing energy consumption and treatment time. Due to its chemical-free nature, it minimizes environmental pollution and adverse effects on protein structure. Additionally, HIU treatment requires simple equipment and reduces operational complexity and energy demand. Its durable devices enable long-term stable operation, thus reducing resource waste and environmental impacts [10]. Currently, several studies have demonstrated that HIU can effectively improve the FC and FS by partially denaturing proteins, leading to more flexible structures and stronger interactions at the water–air interface. For instance, Nazari et al., discovered that HIU enhances the FC of millet protein through cavitation effect [11]. Xiong et al., demonstrated an increase in the FC (from 145.6 % to 200.0 %) of pea protein after HIU treatment, along with an enhancement in FS from 58.0 % to 73.3 % [12]. Similarly, Chen et al., revealed that appropriate applications of HIU induce the unfolding of the protein structure, thereby enhancing the surface hydrophobicity of the egg white solution and contributing to its foaming properties [6]. This increase in FC may be caused by protein denaturation induced by HIU, resulting in a more flexible structure in aqueous solution and stronger interactions at the air–water interface [13].

In addition, temperature has an impact on the foaming properties of proteins. The denaturation temperature of OVA is approximately 84°C. When the processing temperature exceeds or approaches the denaturation temperature, OVA undergoes conformational changes and structural damage, resulting in protein denaturation and loss of its original functional properties [13]. Appropriate heat treatment can enhance the diffusion rate of proteins at the air–water interface, leading to an improvement in foaming properties. For example, Sheng et al., discovered that the FC and FS of OVA increased with rising temperatures within a specific range (20–50 °C). The increase in FC is correlated with the extensibility and flexible structure of hydrophobic proteins. Amphiphilic proteins with high surface hydrophobicity undergo strong adsorption at the air–water interface, resulting in a significant reduction in interfacial or surface tension, thereby facilitating foam formation. However, when the temperature surpassed 60 °C, the FC of OVA gradually declined, accompanied by a sharp decrease in FS [13]. Moreover, Luo et al demonstrated that the foaming properties of OVA exhibited significant improvement upon reaching the optimal heating temperature (50 °C) [14]. These results suggest that temperature is a critical factor in determining protein foaming properties, with appropriate heat treatment contributing to better foaming performance. Despite both HIU and temperature showing beneficial effects on the foaming properties of proteins, recent studies have shown that the combined treatment of heating and HIU exhibited a synergistic effect on the foaming properties of protein. For instance, the simultaneous treatment of HIU and temperature (70, 80, and 85 °C) increased the FC of soy protein (2 % of concentration) increased to 240 % at all studied temperatures, which surpassed the results at room temperature (202 %) [15]. Additionally, the synergistic treatment of HIU and preheating enhanced volumetric viscosity and led to the formation of aggregates through exposing or partially unfolding residues, leading to greater colloidal stability of foam [16]. Therefore, the combined treatment of preheating and HIU techniques may also have a notable beneficial effect on the foaming properties of OVA. However, the underlying mechanism remains to be precisely elucidated.

Therefore, this study aimed to investigate the effects of different pretreatment temperatures and HIU power on the foaming properties of OVA. Subsequently, the FC, FS, and particle size of the treated OVA were evaluated. Additionally, physicochemical parameters including viscosity, surface hydrophobicity, sulfhydryl content, endogenous fluorescence, and zeta potential were measured. Furthermore, structural analysis techniques such as X-ray diffraction (XRD), Fourier transform infrared spectroscopy (FTIR), and scanning electron microscopy (SEM) were employed to gain insights into the mechanisms underlying the improved foaming properties of OVA.

## 2 Materials and methods

### 2.1 Materials

Fresh eggs were purchased from Home Life Supermarket, located in Taigu District, Jinzhong City, Shanxi Province, China. Phosphate buffer, Tris-Glycine buffer, urea, Ellman's reagent, and ethylenediaminetetraacetic acid were obtained from Tianjin Damao Reagent Company (Tianjin, China). Potassium bromide (KBr) and bromophenol blue (BPB) were purchased from Aladdin (Shanghai, China). Polyethylene glycol 8000 (PEG 8000) was sourced from Shanghai Yuanye Biotechnology Co (Shanghai, China).

### 2.2 OVA preparation

The preparation method of OVA was conducted based on the previous method [17]. Initially, the egg whites were separated from the egg yolks and homogenized. PEG-8000 was gradually added at a concentration of 15 % while stirring magnetically at 25°C. After centrifugation at 15000 × g for 15 min, the supernatant was adjusted to pH 4.5. The solution was then centrifuged at 15,000 × g for 15 min, and the precipitate was collected. The precipitate was dissolved in phosphate buffer and the solution was transferred into a dialysis bag. The dialysis was conducted at 4°C for 24 h, with deionized water being replaced every 2 h. Subsequently, the dialysate was subjected to vacuum freeze-drying at 55–60°C for 24 h, and the resulting powder was collected as the OVA product (purity > 95 %).

### 2.3 Preheating treatment

The OVA (5 % w/v) powder was dissolved in distilled water and stirred magnetically for 1 h. The solution was then heated at a constant temperature of 45°C and 60°C in a magnetic stirring water bath for 30 min. Following rapid cooling to room temperature using an ice water bath, the sample was freeze-dried.

### 2.4 HIU treatment

The OVA (5 %, w/v) was dissolved in distilled water and stirred magnetically for 1 h. The OVA solution was then subjected to HIU treatment at different powers (200w, 300w, 400w) and for 20 min each. Meanwhile, the un-HIU-treated and un-preheated treatment groups were used as the control group. During the procedure, the HIU probe was positioned 1.5 cm below the liquid surface, and the entire process was conducted within an ice water bath. The treated samples were subsequently freeze-dried for further analysis.

### 2.5 Determination of FC and FS

The method described by Liu was followed for the determination of FC and FS [18]. 5 mL of protein sample (50 mg/mL) was dissolved in 15 mL of 0.01 M phosphate buffer (pH 9.0). The solution was then transferred into a 50 mL measuring cylinder, and the initial volume of the solution was recorded as V0. Subsequently, the solution was stirred for 1 min using a T25 digital high-speed disperser (ULTRA-TURRAX, IKA, Germany) operating at 20,000 rpm. The foam volume at 2 min was measured and recorded as V1. The foam volume after 2 min and 30 min of standing were measured and recorded as V_1_ and V_2_, respectively. The FC and FS were then determined as follows:

FC=V1-V0V0×100%

FC=V2V1×100%

### 2.6 Determination of viscosity

The viscosity of OVA solutions treated at different temperatures and powers was determined using the NDJ-1 rotational viscometer (L-8800, Hitachi, Japan), following the method described by Xiong et al [19]. The measured solution and foam were transferred into a 50 mL centrifuge tube. The optional rotor was then attached to the connecting rod and gradually submerged into the solution until the level mark on the rotor aligned with the liquid level. After turning on the power, the pointer control lever was pressed and the reading was taken once the pointer stabilized after 30 s. The value indicated by the pointer was recorded as α. The viscosity formula was as follows:

η（mpa·s）=k×α

where k is the coefficient.

### 2.7 Inverted microscopy observation

Freshly stirred foam was uniformly deposited onto a clean glass microscope slide and covered by a coverslip immediately. The morphology of foam samples was subsequently observed under a 10 × eyepiece and a 10 × objective lens using an inverted fluorescence microscope (MCR-302, Anton Paar, Austria) [20].

### 2.8 Determination of surface hydrophobicity

1 mL sample of OVA solution (5 % protein concentration) was mixed with 200 μL of BPB solution (1 mg/mL). The mixture was stirred at 25°C (3000 rpm) for 10 min, then centrifuged at 4°C (2000 rpm) for 15 min. The supernatant was then collected and diluted 10 times with distilled water. The absorbance of the diluted solution was analyzed at 595 nm using a spectrophotometer (TU-1901, Beijing Pu-Analysis General Co., Ltd.). The surface hydrophobicity was then calculated using the formula:

$$\mathrm{BPBbound}\;(\mu g)=200\times \frac{A_{\mathrm{control}}-A_{sample}}{A_{\;\mathrm{control}}}$$

### 2.9 Intrinsic fluorescence analysis

The OVA power was dissolved using 0.01 mol/L phosphate buffer at pH = 7. The fluorescence spectra were obtained using a fluorescence spectrophotometer (RF-5301, Shimadzu, Japan), with untreated OVA solution used as a blank control. The fluorescence spectra were recorded at an excitation wavelength of 280 nm and emission wavelengths ranging from 295 nm to 450 nm A slit width of 5 nm was set for both excitation and emission measurements.

### 2.10 Sulfhydryl groups

For free sulfhydryl content analysis, 0.5 mL of the sample (protein concentration of 10 mg/mL) was mixed with 2.5 mL of Tris-glycine buffer. For the total free sulfhydryl content analysis, 0.5 mL of the sample was added to 2.5 mL of Tris-Glycine buffer containing 8 mol of urea. Then, 40 μL of 4 mg/mL Ellman's reagent was added to each sample. The mixtures were stored for 15 min under light conditions and then analyzed at a wavelength of 412 nm using a UV spectrophotometer (TU-1901, Beijing Pu-Analysis General Co., Ltd.). The amount of sulfhydryl groups (SH) was calculated using the following formula [21]:

$$-SH=\frac{73.53\times D\times A}{C}$$

where: D is the dilution multiple; A is the absorbance value; C is the protein concentration, mg/mL.

### 2.11 Zeta potentials and particle size distribution

5 mL solution of OVA (1 % protein concentration) was prepared using PBS buffer (0.5 mmol/L, pH 7.4). The sample was filtered through a 0.45 μm membrane (Tianjin Jingteng Experimental Equipment Co., Ltd., Tianjin, China) and injected into a transparent zeta potential cuvette with a volume of 0.75 mL, which was then sealed with a cap. Zeta potential and particle size were determined using the Nano-ZS90 instrument (Malvern Instruments Ltd., UK) coupled with an avalanche photodiode. The following testing parameters were used: scattering angle of 90°, equilibration time of 60 s, and testing temperature of 25°C.

### 2.12 FTIR

The sample powder was mixed with KBr in a ratio of 1:100 (w/w) following the method [22]. The mixture was thoroughly ground and then pressed for 2 min at a pressure of 27 MPa, with 3–5 pieces prepared for each sample. Scanning was performed over the range of 4000–400 cm^−1^ with a resolution of 4 cm^−1^. Each sample was scanned with 32 scans, and background subtraction was applied before scanning.

### 2.13 Circular dichroism (CD)

CD spectroscopy using a J-1500CD spectrometer (JASCO Corporation, Japan) was employed to assess the secondary structure of HIU-treated OVA. The measurements of samples (0.2 mg/mL) were conducted in a 0.1 cm quartz cuvette at 25 °C. The scan speed was set at 50 nm/min, with a response time of 4 s and a bandwidth of 1.0 nm. The evaluation of the secondary structure was performed using Yang-Us and JWR software (Jasco International, Tokyo, Japan). The experiment was conducted in triplicate.

### 2.14 XRD analysis

The XRD analysis of the OVA samples under different conditions was performed using an X-ray diffractometer (DX-2700, Dandong Haoyuan Instrument Co., Ltd.). The instrument operates at a wavelength of 0.02°/s and a scanning speed of 2°/min, with a working current and voltage of 40 mA and 40 kV, respectively. The analysis was conducted within the range of 5°-45°.

### 2.15 UV spectrophotometry analysis

The OVA samples (protein concentration of 0.5 mg/mL) were diluted with 20 mM phosphate buffer. Subsequently, the diluted protein solution was transferred into a cuvette for UV–visible spectrophotometry analysis using a TU-190 spectrophotometer (Beijing Pu-Analysis General Co., Ltd.). UV spectra were recorded at a scan rate of 400 nm/min within the range of 200 to 400 nm at 25°C. The control group consisted of 20 mM phosphate buffer containing 0.6 mol/L NaCI. To obtain the second-order derivative of UV spectra, the second-order derivative of the UV spectra (d2A/d2) was calculated.

### 2.16 SEM

The microstructure of OVA was examined using SEM following the methodology outlined by Noskov [23]. A suitable quantity of the sample was directly taken and affixed to a slide using double-sided adhesive. Subsequently, the sample was coated with a thin layer of gold at an angle of 150°. The prepared sample was then observed and photographed at an accelerating voltage of 20 kV using a scanning electron microscope (JSM-5610, Nippon Electron Co., Ltd.).

### 2.17 Statistical analysis

The experiments described above were conducted in triplicate, and the data obtained were presented as mean ± standard deviation. Statistical analysis was performed using SPSS Statistics 21.0 software. The Tukey method was employed to determine significant differences between means (*p* < 0.05). Data visualization and plotting were carried out using Origin 8.5 software.

## 3 Results and discussion

### 3.1 FC and FS of OVA

**Fig 1:**
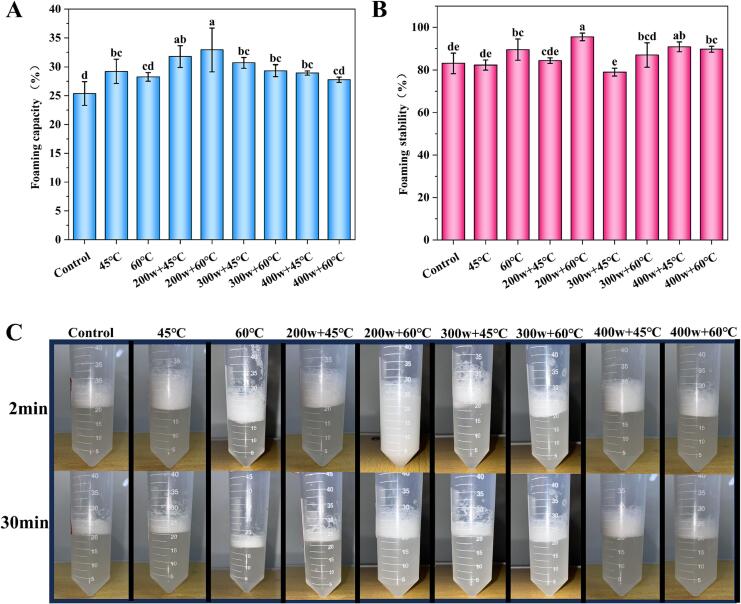
The improvement of foaming capacity (A), foaming stability (B), and foam physical diagrams (C) of OVA after preheating and high intensity ultrasound (HIU) treatment at different powers. Lowercase letters (a-e) denote significant differences between the groups (*p* < 0.05).

Fig. 1A illustrates the effects of preheating temperature and HIU power on the FC of OVA. The results indicate that there is no significant change in FC between 45 °C and 60 °C group (*p* > 0.05). This suggests that preheat treatment at different temperatures did not significantly affect the FC of OVA. Consistent with our findings, Grimaldez et al., investigated the effects of HIU and preheating temperature (50 or 90 °C) on the foaming properties of soy protein and also found that different temperatures had no significant impact on FC [16]. However, the FC of OVA decreases with increasing HIU power at the same temperature. Among them, the highest FC was achieved at 200w + 60 °C, with a value of 32.93 %. Low-power (200w) HIU treatment promoted the dispersion of OVA, enabling the unfolding of folded proteins and exposing hydrophobic groups, thereby reducing interfacial tension and ultimately enhancing FC. However, under high-power (400w) HIU treatment, excessive cavitation effects disrupted the foaming system of the OVA, which caused the dispersed proteins to re-aggregate and bury the hydrophobic groups. Simultaneously, this treatment weakened the interactions between the polypeptide chains of OVA, leading to a decrease in FC [15]. As shown in Fig. 1B, the FS of the 60 °C group (89.50 %) was significantly higher than that of the control group (*p* < 0.05). HIU treatment (200w) significantly enhanced the FS of OVA at 60 °C, with the highest FS of 95.49 %. However, the FS is the lowest at 300w + 45 °C (79.03 %). As depicted in the physical diagrams of foam in each group (Fig. 1C), the highest FC and FS were observed in the 200w + 60 °C group. Hence, the results indicate that 200w + 60℃ treatment can significantly enhance the FC of proteins. HIU facilitates the dispersion of protein molecules within the protein solution, leading to the improvement in FC. HIU treatment leads to increased unfolding of protein molecules, leading to enhanced diffusive adsorption of proteins at the interface. ultimately aiding their diffusion and adsorption at interfaces [24]. In accordance with our findings, Xiong et al., also observed an improvement in the FC of sonicated pea proteins, with values increasing from 145.6 % to 200.0 %. accompanied by an improvement in FS from 58.0 % to 73.3 % [12]. These results revealed that cavitation effects generated by HIU reduce the aggregation of OVA molecules and promote the formation of a more porous protein structure, facilitating its adsorption at the air–water interface. Consequently, this enhances the foaming performance and stability of OVA.

### 3.2 Viscosity and foam morphology of OVA

**Fig 2:**
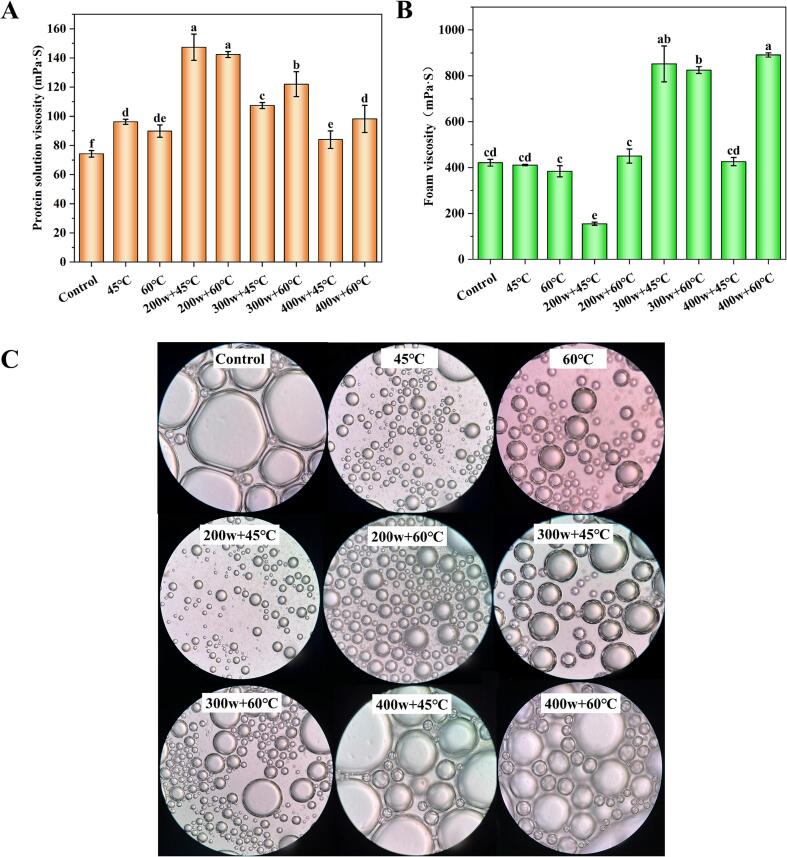
The improvement of protein solution viscosity (A), foam viscosity (B), and foam morphology (C) of OVA after preheating and HIU treatment at different powers.

One of the factors that affect FS is liquid viscosity, and high-viscosity liquids discharge slowly, which helps maintain FS [25]. The viscosity of the solution decreased as the temperature increased during preheating treatment, reaching its maximum of 96.17 mPa·s at 45 °C (Fig. 2A). HIU combined preheating treatment significantly elevated the viscosity of all groups, and the viscosity gradually decreased with increasing HIU power at the same preheating temperature. No significant reduction in solution viscosity was observed as the preheating temperature increased under low-power HIU treatment (200w) (*p* > 0.05). When the HIU power reached 300w or above, higher temperatures resulted in higher viscosities. This indicates that heating at a specific temperature could stimulate protein aggregation through hydrophobic interaction, thus promoting polymerization. As the temperature increased, the intermolecular interaction between proteins showed a tendency for gravitational force to be greater than electrostatic repulsive force, resulting in a gradual increase in the viscosity of the protein system [26]. There was no significant difference (*p* > 0.05) in foam viscosity among the control group (421.25 mPa·s), the 45 °C group (411 mPa·s), and the 60°C group (384 mPa·s) (Fig. 2B). Low-power HIU treatment (200w) did not exhibit a significant enhancement effect on foam viscosity, whereas the combination of 300w or 400w with 60 °C significantly increased foam viscosity. Specifically, the highest foam viscosity was observed in the 400w + 60 °C (891 mPa·s) group.

This suggests that the cavitation effects of high-power HIU treatment alter the structure of OVA, leading to enhanced foam adhesion, flexibility, and improved foam performance [27]. This finding was further supported by the experiments conducted by NA Mir, which demonstrated an improvement in foaming properties of the protein from 42 % to 76 % after 25 min of treatment compared to only 5 min of HIU treatment. Consequently, HIU exhibited significantly higher adsorption kinetics at the air–water interface, resulting in foams with smaller bubble diameters, increased spillover, stability, and yield stress, as well as reduced liquid drainage [28]. The microscopy images of foams subjected to various treatment conditions revealed that the preheating-treated OVA samples exhibited numerous small-sized foams with higher foam density but a non-uniform distribution of small and large foams compared to the untreated OVA (Fig. 2C). HIU reduced foam size, with the 200w + 60 °C group significantly reducing foam size while maintaining uniformity. However, an increase in HIU power (400w) resulted in significantly larger and unevenly distributed foams compared to the groups treated with 200w. It has been demonstrated that foam size is closely related to foaming properties, and larger foam size may lead to decreased FS [27]. The cavitation effect induced by low-power HIU treatment (200w) enhances the dispersion of protein structure, resulting in the unfolding of more folded proteins and exposing previously hidden hydrophobic groups [29].

### 3.3 Interfacial properties of OVA

**Fig 3:**
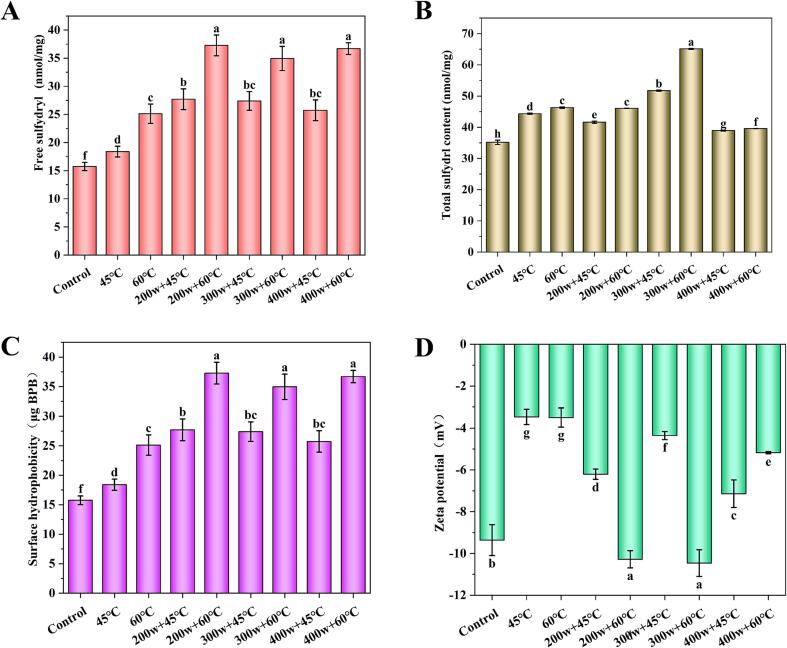
The improvement of free sulfydryl (A), total sulfydrl content (B), surface hydrophobicity (C), and zeta potential of OVA after preheating and HIU treatment at different powers.

The sulfhydryl groups are crucial for maintaining the stability and functional properties of OVA. The preheating treatment noticeably increased the concentration of free sulfhydryl groups in OVA. The higher the temperature, the greater the concentration of free sulfhydryl groups (Fig. 3A). Thermal treatments cause protein denaturation, thereby exposing sulfhydryl groups and elevating the sulfhydryl content [30]. However, the power levels of HIU did not significantly impact the sulfhydryl content of OVA at the same temperature. However, at 60°C, HIU caused a noteworthy rise in free sulfhydryl content, reaching its peak at 37.26 nmol/mg in the 200w + 60°C group. The cavitation effect induced by HIU has been shown to partially unfold protein molecules, exposing previously hidden free sulfhydryl groups from the hydrophobic interior to the outer surface. As illustrated in Fig. 3B, the total sulfhydryl content exhibited an increase corresponding to the rise in preheating temperature. Moderate power HIU treatment (300w) significantly increased the total sulfhydryl content compared to the group without HIU treatment. Notably, the highest total sulfhydryl content was observed in the 300w + 60 °C group, reaching 65.11 μmg/g. However, both low-power and high-power HIU treatments did not show a significant enhancement in total sulfhydryl content. HIU treatment induces changes in protein conformation and unfolding of the internal protein structure, thus exposing internal sulfhydryl groups [13]. It can be inferred that HIU potentially enhances the exposure of sulfhydryl groups and disrupts disulfide bonds within the protein structure, leading to reduced molecular stability and an increase in free sulfhydryl content [31].

Both preheating alone and combined with HIU treatment significantly enhanced the surface hydrophobicity compared to the control group. Among them, the highest surface hydrophobicity was found in the 200w + 60 °C group (37.28 μg BPB) (Fig. 3C). This finding suggests that heat treatment facilitates the unfolding of the water-soluble globular protein structure, thereby exposing internal hydrophobic groups and improving the interfacial properties of the protein [32]. Additionally, the high shear energy waves generated by HIU can induce protein molecules to unfold, thereby reducing intermolecular aggregation and exposing hydrophobic residues within the internal regions to the external environment. The reduction in protein molecular aggregation increases the total surface area of the particles, further enhancing surface hydrophobicity [33]. Hydrophobic interactions are crucial for maintaining the tertiary structure of proteins and are essential for the stability, conformation, and functionality of natural proteins. Surface hydrophobicity reduces barriers to protein adsorption at the water–air interface. Looser protein structures and the exposure of peptide chains expose more hydrophobic cavities and groups, facilitating enhanced adsorption to the interface. This facilitates improved foaming properties of isolated proteins [34]. Therefore, the increased surface hydrophobicity of OVA after preheating combined with HIU treatment contributes to its effervescence.

Surface groups of proteins in solution undergo ionization, resulting in positive charges in acidic solutions and negative charges in alkaline solutions [35]. Compared to the control group, the absolute value of zeta potential significantly decreased after preheating treatment (Fig. 3D). This indicates that higher temperature induces the rearrangement of charged amino acids on the surface of protein molecules, thereby altering their surface charge. Consequently, hydrophobic amino acids of OVA were exposed, resulting in a decrease in the number of surface charges on the molecule [36]. Furthermore, the absolute values of zeta potential treated with 200w + 60 °C and 300w + 60 °C were significantly higher than the control group. Specifically, OVA in the 300w + 60 °C group exhibited a maximum negative potential value of −10.46 mV. This suggests that HIU treatment disrupted the aggregation of protein molecules, exposing more negatively charged amino acids and increasing the overall charge and potential values [21]. Moreover, the lower surface charge weakens the electrostatic repulsion between molecules, leading to their aggregation and precipitation formation, which ultimately results in instability of the solution system [37].

### 3.4 Particle size of OVA

**Fig 4:**
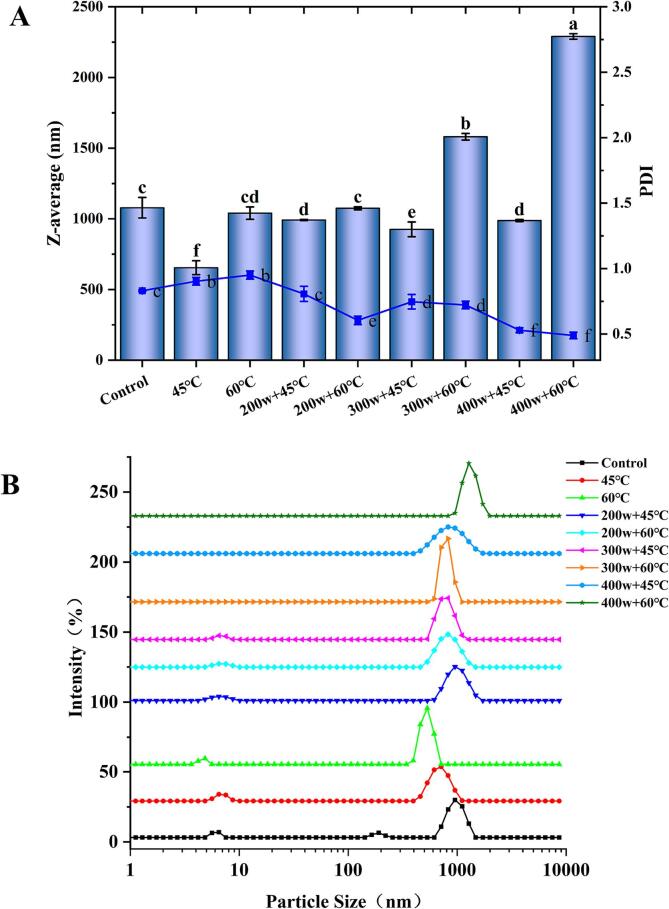
The Z-average (A) and particle size distribution (B) of OVA after preheating and HIU treatment at different powers.

Studies have shown that smaller protein molecules diffuse faster at the air–water interface and exhibit superior foaming properties [38]. As depicted in Fig. 4A, the diameter of OVA particles varies from 654.3 nm to 2290 nm. The group treated at 45 °C exhibited a significant reduction in the particle size of OVA, with the smallest average size of 654.3 nm. This decrease in average particle size can be attributed to the temperature being close to or above the denaturation temperature of OVA. As a result, the molecular motion of protein molecules becomes more intense, increasing the likelihood of intermolecular interactions and forming aggregates through hydrophobic interactions. These aggregates dissociate at higher temperatures, leading to a decrease in the average particle size [25]. Furthermore, the combined treatment of 45 °C and HIU resulted in a significant decrease in particle size compared to the control group. However, it displayed a noticeable increase in particle size compared to the treatment at 45 °C alone. This can be attributed to the cavitation effect induced by HIU treatment, which disrupts aggregates and reduces particle size through powerful shear forces [39]. However, the largest OVA particle size was observed after treatment at 400w + 60 °C. This may be due to the cavitation effect induced by HIU treatment, which instantly generates higher temperatures, leading to the aggregation of OVA molecules [40].

The polymer dispersity index (PDI) is employed to characterize the distribution of molecular weights in polymers. A higher PDI value indicates a wider range of molecular weights, whereas a lower PDI value suggests a more uniform distribution. It is evident that the preheating treatment significantly enhanced the PDI value compared to the control group. The results indicate that the molecular weight distribution of OVA becomes broader and the system becomes unevenly distributed after heating. In contrast, HIU at 200w + 60°C treatment resulted in a decrease in PDI of OVA, leading to a more uniform molecular weight distribution. Moreover, both the individually preheated samples and the samples treated with a combination of preheating and 300w HIU exhibit a leftward shift in the particle size distribution curve (Fig. 4B). This indicates that HIU treatment effectively disperses the agglomerated macromolecular protein particles. The strong shear forces generated by ultrasonic cavitation might hinder the aggregation of OVA molecules, thereby promoting dispersion [41].

### 3.5 Structural properties of OVA

**Fig 5:**
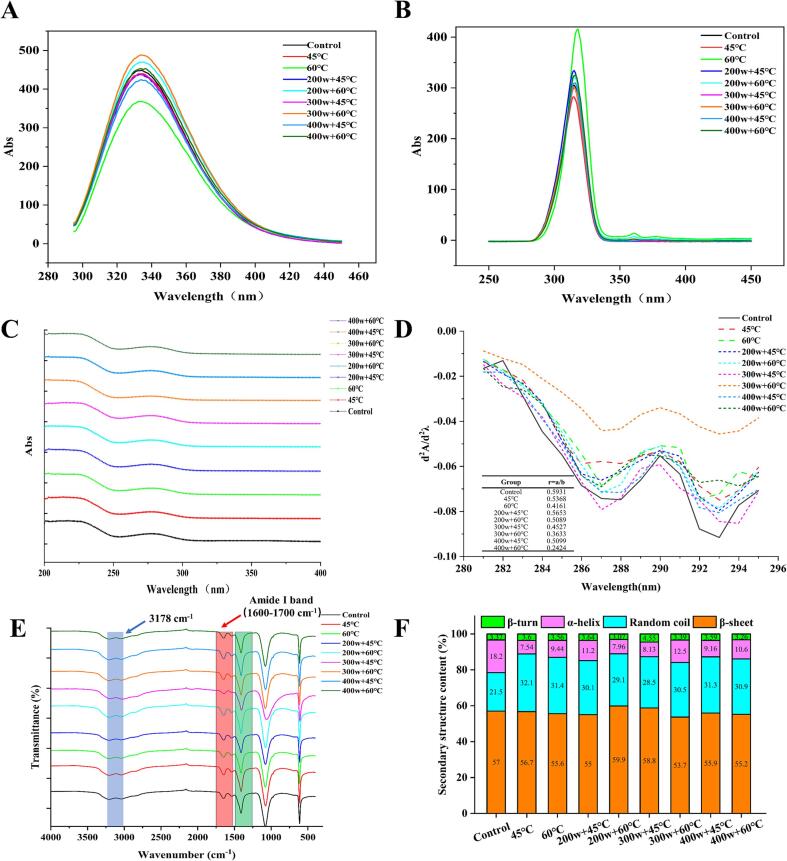
The structure analysis of OVA after preheating and HIU treatment at different powers. (A). Emission intrinsic fluorescence; (B) synchronous intrinsic fluorescence; (C). Ultraviolet spectrum; (D). Second-order derivative of UV; (E) Fourier transform infrared spectroscopy; (F). Secondary structure content.

Fluorescence spectroscopy is commonly utilized in the investigation of conformational changes in proteins, with endogenous fluorescence predominantly originating from aromatic amino acids, such as tryptophan and tyrosine. The alterations in their surroundings and hydrophobicity can be assessed through concurrent fluorescence spectroscopy [25]. As depicted in Fig. 5A, all groups exhibited the highest fluorescence intensity at 340 nm when excited at a wavelength of 280 nm. Compared to the blank group, the fluorescence intensity of the OVA subjected to preheating at 60°C showed a slight reduction, whereas the group treated with HIU demonstrated a significant enhancement in fluorescence intensity. Particularly, the group subjected to 300w + 60°C exhibited the highest emission fluorescence intensity. This observation can be attributed to the modification in protein aggregation induced by HIU treatment, leading to an increased exposure of tryptophan to a more accessible microenvironment that impacts the tertiary structure of the protein [28]. Furthermore, all groups exhibited the highest fluorescence intensity at 320 nm with an excitation wavelength of 230 nm (Fig. 5B). The fluorescence peak of the 60°C group exhibited a red shift compared to the blank group, indicating the formation of a large number of soluble aggregates between molecules due to the temperature increase [29]. These aggregates possess specific optical properties due to their molecular arrangement and intermolecular interactions. Conversely, HIU treatment caused a slight blue shift in the fluorescence peak of OVA (from 320 to 310 nm). This shift can be attributed to the cavitation effect induced by HIU treatment, which alters the microenvironment surrounding tryptophan and increases hydrophobicity, leading to a blue shift in the maximum emission wavelength [42].

The changes in both the microenvironment of amino acids and the structure of proteins can be reflected by the variations in UV absorption spectra. As depicted in Fig. 5C, the combination of preheating and HIU treatment led to a significant increase in the absorbance of OVA compared to the control group. Among them, the 200w + 60 °C group had the highest absorbance value, indicating a significant impact on the structure of OVA. UV second derivative spectra enhance UV absorption spectrum resolution and reduce overlap caused by aromatic amino acids, thus providing more valuable information about the alterations in the microenvironment of amino acid residues [43]. As illustrated in Fig. 5D, the sets of second derivative spectra exhibit two peaks at 283 nm and 291 nm, as well as two valleys at 287 nm and 293 nm. The peak at 291 nm is a result of the combined impact of tyrosine and tryptophan residues. Additionally, the absorption peak of OVA at 283 nm undergoes a redshift after preheating and HIU treatment. This phenomenon suggests that the alteration in tertiary conformation of the protein may be induced by the heating and HIU, which affects the microenvironment of the protein molecule and leads to the relocation of amino acid residues to a more hydrophobic environment [44]. To evaluate the changes in the microenvironment of tyrosine residues, the ratio of the distance between the positive and negative absorption peaks, known as the amplitude ratio (r), was calculated by dividing the second-order derivative absorption peak by the valley (r = a/b) [45]. After the heating treatment, the r values decreased significantly compared to the blank group. Similarly, the r value decreased with higher HIU power, indicating that the microenvironment of tyrosine residues became more hydrophobic, which aligns with the results of surface hydrophobicity. After the heating treatment, the r values decreased, and at 60 °C, they were significantly lower compared to the blank group. Similarly, the r value decreased as the HIU power increased. This indicates that the microenvironment of tyrosine residues became more hydrophobic with the preheating and HIU treatment, consistent with the results of surface hydrophobicity.

In the FTIR spectra of OVA (range of 4000 to 500 cm^−1^), a weak absorption peak was observed near 3178 cm^−1^, which was attributed to the hydrogen bonds and O-H stretching vibrations in the protein matrix (Fig. 5D). The amide I band (1600–1700 cm^−1^) exhibits strong infrared absorption, providing rich secondary structure information mainly derived from the stretching and bending vibrations of C = O bonds. Characteristic absorption peaks in the amide I region are observed in all groups. Compared to the control group, the peak intensity of the amide I band in the 200w treated group significantly increases, while the peak position remains unchanged. The peak intensity of the amide III band (1430–1240 cm^−1^) in the 200w + 60°C and 400w + 45°C groups also showed significant increases compared to the control group. Fig. 5F reveals the secondary structure content of OVA under different treatment conditions. It is evident that the secondary structure of OVA is primarily composed of β-folds, random coil, and α-helix. After preheating at 45°C, the α-helix content of OVA significantly decreased from 18.2 % to a minimum of 7.54 %, while the content of irregular coils notably increased to 32.1 %. Preheating alone had no significant effect on the β-sheet content, but the 200w + 60°C and 300w + 45°C groups exhibited a significant increase in both β-sheet and irregular curl content, while also showcasing a noteworthy decrease in α-helix content. The decrease in α-helix content suggests the denaturation of OVA molecules during treatment, while the increase in β-sheet content indicates the rearrangement of denatured molecules and the generation of more stable products [23]. HIU treatment was shown to disrupt hydrogen bonds within the protein molecule, exposing the hydrophobic region and promoting partial conversion of α-helix structures to β-folds by hydrogen bonding [46]. The abundance of β-folding and irregular curling enhanced the flexibility of the OVA molecule and loosened the protein molecular structure, facilitating interfacial adsorption and thereby enhancing foaming properties to a certain extent [36].

### 3.6 The crystalline properties of OVA

**Fig 6:**
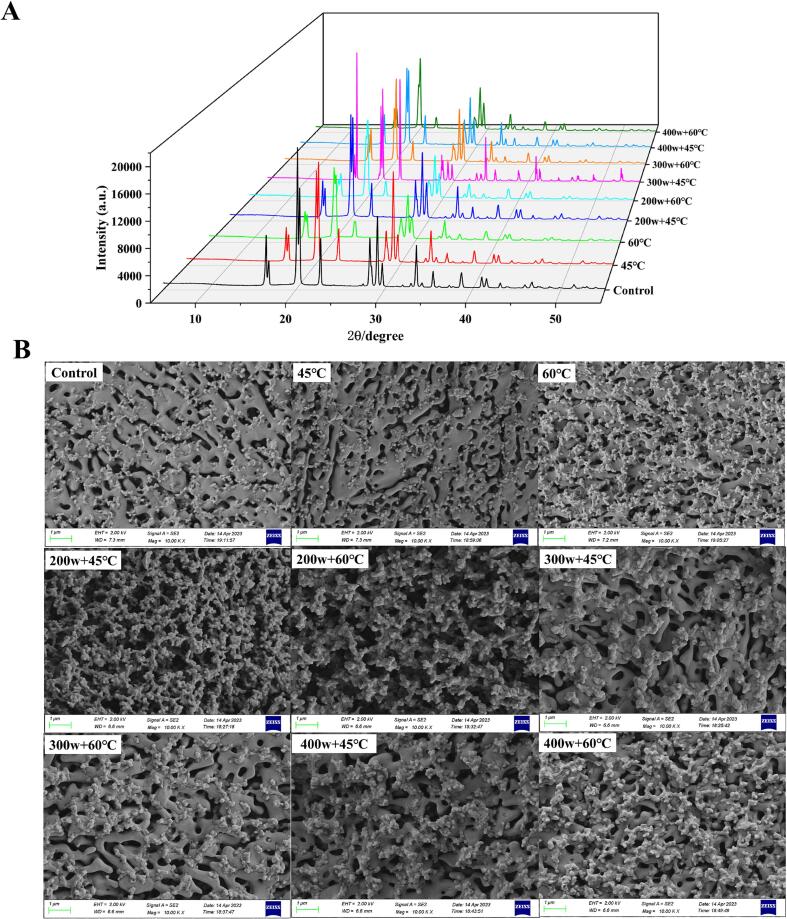
The XRD (A) and microstructure (B) of OVA after preheating and HIU treatment at different powers.

The crystalline properties of OVA were examined using XRD spectroscopy. XRD is commonly employed to investigate the crystallinity and structural characteristics of crystals. In general, sharp peaks in the XRD pattern indicate a crystalline structure, while broad peaks are indicative of an amorphous structure [44]. OVA exhibits two distinct characteristic diffraction peaks at 2θ = 20° and 30° (Fig. 6A). After heating treatment, the peak intensity of OVA decreased and became less pronounced at 20° compared to the control group, suggesting structural damage caused by heating. Conversely, preheating combined HIU-treated OVA exhibited increased peak intensity and sharper peaks at 20°, which closely resembled the peak of the control group. This suggests that HIU treatment helps preserve the crystal structure of OVA. In the rapid mixing of protein solutions, a large amount of gas was introduced to form an air–water interface. This interface adsorbs OVA from the solution through the interactions between peptide chains and strengthens the interfacial film by forming a two-dimensional protective network, promoting the formation and stability of foam [13]. Notably, the highest and sharpest OVA diffraction peak intensity was observed under the 200w + 45 °C treatment, indicating that HIU promoted the formation of an ordered and stable OVA structure. Such structural changes lead to a more favorable unfolding degree of the internal structure and improved viscoelastic properties of the film formed at the air–water interface of bubbles [12]. These modifications ultimately enhance the foaming properties of the protein.

### 3.7 Micromorphology of OVA

As illustrated in Fig. 6B, OVA molecules in the control group exhibited small size, relatively uniform distribution, and a smooth surface. However, evident aggregation of protein molecules could be observed after the heating treatment, with the degree of aggregation increasing with higher temperatures. For instance, the originally smooth surface became uneven and the OVA molecules aggregated at 60°C. The surface of OVA became rougher, and the appearance of uniform pore-like structures with aggregated particle formations was observed after preheating combined with HIU treatment. Particularly, the pore-like structures showed a consistent distribution accompanied by particle aggregation after 200w HIU treatment. Guo et al., demonstrated that these microscopic morphological changes might be attributed to the disruption of hydrogen bonds and van der Waals forces between protein molecules caused by shock waves and shear forces during the collapse of cavitation bubbles during HIU treatment [30]. This disruption leads to the breakdown of cross-linkages between protein molecules. The formation of this loose pore-like structure exposes hydrophobic groups, thereby increasing hydrophobicity. Consequently, the foaming properties of isolated proteins can be enhanced [47].

### 3.8 Pearson correlation analysis and involved mechanism

**Fig 7:**
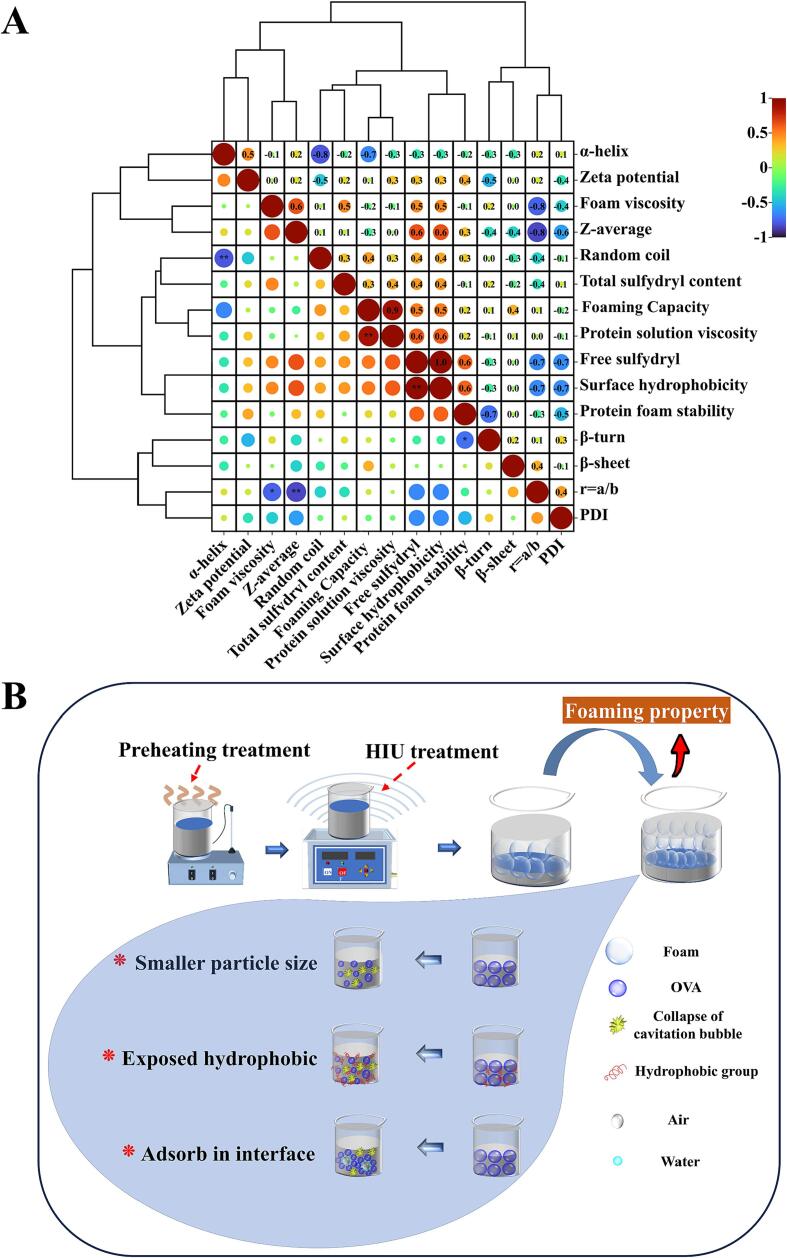
The Pearson correlation analysis (A) and mechanism diagrams of foam formation (B) of OVA after preheating and HIU treatment.

The Pearson correlation analysis was conducted to examine the correlation between foaming properties and various physicochemical structural properties of OVA after preheating and HIU treatment. The color scale correlation analysis graph provided an intuitive representation of the correlation between foaming properties and various physicochemical structural properties. The color scale correlation analysis reveals that FC and protein viscosity (correlation coefficient 0.9), surface hydrophobicity and free sulfhydryl group (correlation coefficient 1.0) were displayed in dark red, indicating a significant positive correlation between these factors (Fig. 7A). Furthermore, there was a negative correlation (-0.8) identified between r = a/b and average particle size, as well as the content of random coil and α-helix. Similarly, a negative correlation (-0.7) was evident between foam stability and β-turn content, all represented by the blue blocks in the graph.

These findings demonstrate that the combination of preheating and HIU treatment significantly improves the foaming properties of OVA by enhancing its structural and physicochemical properties, as illustrated in Fig. 7B. This treatment reduces the particle size of OVA, facilitating its adsorption onto the air–water interface and promoting rapid and uniform dispersion, thereby enhancing the FC of OVA. Simultaneously, the increased specific surface area resulting from the smaller particle size exposed more hydrophobic groups and partially unfolded the protein structure. This accelerates protein–protein interactions, facilitates adsorption onto the air–water interface, and forms a thin viscoelastic layer, ultimately improving FS.

## 4 Conclusion

The objective of this experiment was to reveal foaming properties enhancement of OVA after preheating and HIU treatment from physicochemical properties and conformational analysis and explore the underlying mechanisms. The results revealed that OVA exhibited the highest FC and FS in the 200w + 60°C group. Additionally, significant improvements were observed in the content of free sulfhydryl groups, solution viscosity, and surface hydrophobicity. The disruption of protein aggregation caused by the cavitation effect of HIU led to the exposure of negatively charged amino acids, smaller particle sizes, and continuous uneven pore structure. In conclusion, the preheating and HIU treatment offers a novel approach to enhance the foaming properties of OVA and has potential future applications in the food industry.

## CRediT authorship contribution statement

Zhihui Yu: Conceptualization, Data-acquisition, Writing – original draft. Li Ma: methodology, Writing – original draft. **Binbin Liu:** Data-acquisition, Editing – original draft. **Wenqing Wang:** Data-acquisition, editing-original draft. **Ziqi Shang:** Data-acquisition, Editing-original draft. **Huichao Dang:** Conceptualization, Data-acquisition. **Chunyou Liu:** Conceptualization, Review & editing, Data curation, Formal analysis, Supervision.

## Declaration of Competing Interest

The authors declare that they have no known competing financial interests or personal relationships that could have appeared to influence the work reported in this paper.
